# The Impact of COVID-19 on the Adaptive Functioning, Behavioral Problems, and Repetitive Behaviors of Italian Children with Autism Spectrum Disorder: An Observational Study

**DOI:** 10.3390/children8020096

**Published:** 2021-02-02

**Authors:** Martina Siracusano, Eugenia Segatori, Assia Riccioni, Leonardo Emberti Gialloreti, Paolo Curatolo, Luigi Mazzone

**Affiliations:** 1 Department of Biomedicine and Prevention, University of Rome Tor Vergata, Via Montpellier 1, 00133 Rome, Italy; leonardo.emberti.gialloreti@uniroma2.it; 2 Department of Biotechnological and Applied Clinical Sciences, University of L’Aquila, Via Vetoio 40, 67100 L’Aquila, Italy; 3 Child Neurology and Psychiatry Unit, Systems Medicine Department, University of Rome Tor Vergata, Via Montpellier 1, 00133 Rome, Italy; eugeniasegatori210@gmail.com (E.S.); assiariccioni@gmail.com (A.R.); curatolo@uniroma2.it (P.C.); luigi.mazzone@uniroma2.it (L.M.)

**Keywords:** coronavirus, lockdown, parents, behavior, adaptive, autism spectrum disorder, online, COVID-19, pandemic

## Abstract

Children with autism spectrum disorder (ASD) and their families have represented a fragile population on which the extreme circumstances of the COVID-19 outbreak may have doubly impaired. Interruption of therapeutical interventions delivered in-person and routine disruption constituted some of the main challenges they had to face. This study investigated the impact of the COVID-19 lockdown on adaptive functioning, behavioral problems, and repetitive behaviors of children with ASD. In a sample of 85 Italian ASD children (mean age 7 years old; 68 males, 17 females), through a comparison with a baseline evaluation performed during the months preceding COVID-19, we evaluated whether after the compulsory home confinement any improvement or worsening was reported by parents of ASD individuals using standardized instruments (Adaptive Behavior Assessment System (Second Edition), Achenbach Child Behavior Checklist, Repetitive Behavior Scale-Revised). No significant worsening in the adaptive functioning, problematic, and repetitive behaviors emerged after the compulsory home confinement. Within the schooler children, clinical stability was found in reference to both adaptive skills and behavioral aspects, whereas within preschoolers, a significant improvement in adaptive skills emerged and was related to the subsistence of web-delivered intervention, *parental work continuance*, and online support during the lockdown.

## 1. Introduction

The 2019 coronavirus disease (COVID-19) spread around the world from December 2019 initiating a pandemic that is still in effect. COVID-19 severely impacted the health and the wellbeing of citizens and countries worldwide and determined a correlative impairment of finances and economics with a consequent derangement of everyday life’s scheduling. At the beginning of the pandemic, Italy represented one of the most affected by the virus among European countries. In order to reduce the spreading of the infection, the Italian government imposed home-confinement for all residents from 9 March 2020 to 4 May 2020. Schools, restaurants, shops, museums, and gyms were compulsorily closed. Most of the work activities were interrupted except for those considered as essential (i.e., doctors, nurses, employed in supermarkets). Home exiting was permitted only for indispensable and critical needs (i.e., hospital, supermarket, shops for personal care and hygiene). After 4 May 2020, in the re-opening phase, citizens were allowed to leave their homes, but they had to observe social distancing and wear safety devices to reduce the risk of infection.

While the lockdown has been a challenge for all citizens in general, in consideration of the impact on social and economic matters, the home confinement represented a particularly hazardous double dare for individuals with autism spectrum disorder (ASD) and their families. In fact, under such extreme circumstances, people with ASD faced additional difficulties related to a condition already characterized by impairment in social communication, restricted interests, and repetitive behaviors associated with reduced tolerance of changes [1]. The above-mentioned additional difficulties may sum up to difficulty in understanding the situation (reduced or lack of abstract reasoning), difficulty in assuming the consequences of unsafe behavior (not wearing masks leads to an increased possibility of infection), routine disruption, interruption of all in-person interventions [2]. Caregivers of autistic children had to face the challenges strictly related to the COVID-19 outbreak and those concerning the guidance and handling of their children. As a result, a worsening of ASD clinical features—with special regards to the ones concerning behavior—was plausibly expected after the lockdown [2]. Together with autism core symptoms, medical comorbidities such as sleep disorders, frequently described in ASD individuals [3], may also have represented an additional challenge in such extreme circumstances. Greater sleep problems have, in fact, been reported during home confinement, and associated with more severe autism symptoms [4]. Asbury et al. [5] qualitatively measured, using a free-response question, the effect of the COVID-19 outbreak on the mental health of children with special educational needs and disabilities (SENDs)—including ASD individuals—and on their caregivers. The results of the study showed that most of SENDs parents described themselves as overwhelmed, and only a few of them reported no impact or any improvement in their children [5].

Furthermore, Colizzi et al. [6] conducted a parent-survey on 527 ASD individuals (mean age 13 years old), reporting a behavioral problem increase in one out of three of the sample after the COVID-19 outbreak. Moreover, they found that the presence of disruptive behaviors preceding the pandemic was related to a worse outcome. Concordant with this, a Turkish study [7] on children and young adults with autism reported increased stereotypies, aggression, hypersensitivity behavioral problems, sleep, and appetite alterations after the pandemic, describing in their sample clinical symptoms similar to post-traumatic stress disorder. However, to our knowledge, neither study employed standardized quantitative tools—administered before and after the lockdown—in order to measure the actual behavioral outcome of the children with ASD.

The aim of this study was to investigate in a sample of Italian autistic individuals, any change in adaptive functioning and in repetitive and behavioral (internalizing and externalizing) problems, appearing after the compulsory home confinement, through a comparison of the data collected during the pandemic with evaluations performed before the COVID-19 outbreak. We also aimed to evaluate if the outcome following the lockdown was associated with child and parental variables such as subsistence of child therapeutic intervention in remote modality, *online parental support*, and *parental work continuance* during home confinement.

## 2. Methods

### 2.1. Participants

The study was approved by the local institutional review board (IRB) of the University of Rome Tor Vergata Hospital (Register Number # 216.20). Parents of all participants gave written informed consent. Our sample was constituted by children with ASD coming from the clinical database of the Child Psychiatry Unit of the University of Rome Tor Vergata Hospital (for study protocol and Strobe Checklist see Supplementary Materials: Tables S1 and S2). Recruitment was performed during March–April 2020. Overall, 335 individuals were detected from the database by a multidisciplinary team of psychiatrists and psychologists and considered for eligibility. In order to be eligible, participants were required to have a diagnosis of ASD according to the Diagnostic and Statistical Manual of Mental Disorders (Fifth Edition) (DSM-5) [1], (supported by the assessment of the Autism Diagnostic Observation Schedule (Second Edition) (ADOS–2) [8] by a licensed clinician), an age in the range of 2–18 years old, and to have undergone a behavioral intervention before the COVID-19 outbreak. 

**Figure 1:**
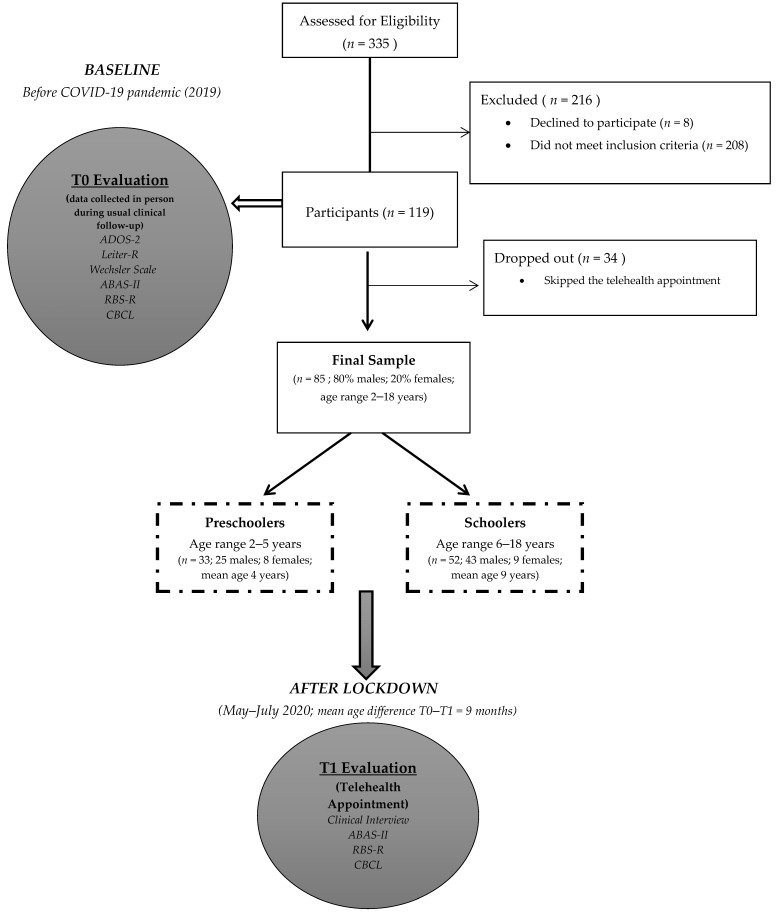
Flowchart of the study. Illustrated in the Figure are the main methods of the study (participants, procedure, and materials).

Finally, 119 participants were considered eligible for the research (208 did not meet inclusion criteria). The multidisciplinary team of our unit contacted the families by phone, described the study, and invited them to participate, planning a telehealth appointment (eight declined to participate). A total of 34 participants dropped out of the study (they skipped the telehealth appointment). Therefore, the final sample consisted of 85 participants (80% males; 20% females; age range 2–18 years old; 33 preschoolers, 52 schoolers) (Figure 1). This study mainly included individuals originating from the central-south of Italy (regions less affected by COVID-19 at the time). 

### 2.2. Procedure

All ASD participants included in the study had been clinically assessed before the pandemic in 2019 in the context of a regular clinical follow-up performed in the Child Psychiatry Unit of the University of Rome Tor Vergata, by a multidisciplinary team (child psychiatrists, psychologists). In particular, the baseline clinical evaluation (T0) performed in-person, included an assessment of autistic symptoms, adaptive functioning, behavioral problems, and repetitive behaviors through the administration of standardized instruments (see the paragraph below). Furthermore, the intelligence quotient (IQ) was measured for the majority of participants.

After the compulsory home-confinement, in the re-opening phase (T1), from May 2020 to July 2020, within a mean distance of 9.5 months from baseline, ASD children included in the study and their parents underwent a planned telehealth appointment with a child psychiatrist of the University of Rome Tor Vergata Hospital, because the restrictions did not allow to perform an in-person clinical evaluation where the safety distance could not be guaranteed. Specifically, parents were administered the same standardized measures evaluating adaptive functioning, repetitive behaviors, and behavioral problems. The assessment of autistic symptoms and IQ of ASD participants was clearly not performed over telehealth appointment. A clinical interview was conducted on the parents for the purpose of evaluating the main routine disruption and the environmental changes that occurred during the pandemic, with a special focus on the job condition and the children’s therapeutical intervention. Specifically, by the clinical interview, we investigated whether during lockdown ASD children continued their usual behavioral intervention in remote modality and with a frequency of at least once a week (variable named “*online child intervention*”); whether parents received at least a weekly online psychoeducational support in order to be helped face their children’s main and overall difficulties due to the emergency situation (variable named “*online parental support*”); if the parent with a stable job, continued to be employed during the lockdown, either remotely or in-person (variable named “*work continuance*”).

It is necessary to specify that the children’s behavioral intervention and the online support addressed to parents during the lockdown were not delivered by our unit, but were included in the usual therapeutical intervention. Therefore, no homogeneity in duration, frequency, and contents can be guaranteed regarding both variables (“*online child intervention* and *online parental support*”).

### 2.3. Materials

#### 2.3.1. Cognitive and Adaptive Functioning Measures

The intelligence quotient (IQ) of participants was evaluated at baseline through the Leiter International Performance Scale-Revised [9], the Wechsler Preschool and Primary Scale of Intelligence (Third Edition) (WPPSI-III) [10], or the Wechsler Intelligence Scale for Children (Fourth Edition) (WISC-IV) [11]. The cognitive measure was chosen on the basis of age, expressive language level, and cooperation of each participant. All of these measures used the same standard scores (SS = 100) and standard deviations (SD = 15).

On the basis of the IQ value, we dichotomized the sample in “intellectual disability” (ID) (IQ ≤ 70) and “no intellectual disability” (No ID) (IQ > 70) (Table 1).

In order to evaluate the participants’ adaptive functioning, the Adaptive Behavior Assessment System (Second Edition) (ABAS-II) [12], a parent-report checklist, was employed. Parents of all participants were administered the “0–5 years” or the “5–21 years” form, depending on the child’s age. The child’s ability to implement an activity is rated (from 0 = “not able to do” to 3 = “able to do it and always performs it when needed”) in relation to ten adaptive skill areas (communication, use of the environment, preschool competences, domestic behavior, health and safety, play, self-care, self-control, social abilities, and motility). These functioning areas are grouped in three main adaptive domain scores—(1) conceptual (CAD), (2) practical (PAD), and (3) social (SAD). In addition, a general adaptive composite (GAC) score—a comprehensive domain of the adaptive scale- is computed by the sum of scaled scores from the 10 skill areas. Raw scores are converted in scaled and finally in a standardized composite score, with a population mean of 100 and a standard deviation of 15. For the statistical analyses, composite scores of the three adaptive domains (CAD, PAD, and SAD) plus GAC were used.

#### 2.3.2. ASD Diagnostic Measure

The ADOS-2 [8], which is performed by a licensed clinician, was employed in order to confirm participants’ ASD diagnosis. The ADOS-2 is a semi-structured observational assessment measuring current autistic symptoms, including socio-communicative difficulties and repetitive and restricted behavior. The ADOS-2 is divided into different modules. Each module is aimed at a specific level of expressive language ability (ranging from pre-verbal to fluent speech). The choice of modules is based on the participant’s age and expressive language level. In the present study, participants were administered different modules (Module 1 to 4) according to their age and expressive language level. In order to compare scores across different modules, the ADOS-2 calibrated severity score (CSS) was calculated for each participant. The CSS, ranging from 1 to 10, identifies four different categories (none, mild, moderate, and high) and provides a measure for the level of autism severity.

#### 2.3.3. Repetitive Behavior and Restricted Interests Assessment

Participants’ repetitive behaviors and restricted interests were assessed by a self-reported scale completed by caregivers, the Repetitive Behavior Scale-Revised (RBS-R) [13]. In our study, we employed the Italian version of RBS-R [14]. The RBS-R questionnaire consists of 43-items, grouped in six subscales (stereotypic behavior, self-injurious behavior, compulsive behavior, ritualistic behavior, sameness behavior, and restricted interests behaviors) rating repetitive behaviors on a four-point Likert scale (ranging from 0 to 3) depending on the frequency and severity of the behavior. The five-factor solution was used for the scoring [15]. The five-factor solution implies that the “ritualistic behavior and sameness behavior” subscales are integrated into one subscale named the “ritualistic/sameness behavior.” The raw score of each subscale was calculated by adding all the items provided for the scoring. Finally, the sum of all five subscales scores (RBS Total) was calculated.

#### 2.3.4. Problematic Behavior Measure

Emotional symptoms and behavioral problems of ASD children and youth were assessed using the questionnaire Achenbach Child Behavior Checklist (CBCL) [16]. According to the age participants, parents were administered the “18 months–5 years” or the “6–18 years” form. Caregivers were asked to rate their child adverse behavior on a three-point Likert Scale (0 = not true, 1 = sometimes true, and 2 = often true), depending on the frequency of the behavior, with a higher score showing more problematic behavior. According to the T-scores, the behavior is considered as typical (*T* < 65), borderline (*T* = 65–69), and clinically significant (*T* ≥ 70).

The “18 months–5 years” form consists of 110 items organized in seven syndrome scales (emotionally reactive, anxious/depressed, somatic complaints, withdrawn, sleep problems, attention problems, and aggressive behavior). Each scale is organized into two main domains—internalizing and externalizing symptoms. Moreover, a total behavior score can be calculated.

The “6–18 years” form consists of 113 items grouped in eight syndrome scales (anxious/depressed, withdrawn/depressed, somatic complaints, social problems, thought problems, attention problems, rule-breaking behavior, and aggressive behavior). In addition, in this case, two main domains—internalizing and externalizing symptoms—and a total score are provided. For the purpose of this study, employed scales were the internalizing and externalizing symptoms scales in association with the total score of both CBCL forms. 

## 3. Statistical Analyses

The two subgroups of preschoolers and schoolers have always been analyzed separately. Changes in ABAS-II, RBS-R, and CBCL scores between T0 and T1 (pre- and post-home confinement due to the COVID-19 pandemic) were evaluated with the paired sample *t*-test. Comparisons between groups in terms of ABAS-II, RBS-R, or CBCL score differences between T0 and T1 have been analyzed through the independent sample *t*-test. Spearman’s correlations were used to evaluate the relations between quantitative variables. Two-way ANOVA with tests of between-subjects effects was used to test for possible interactions between independent variables, such as the presence of ID and sex; *online child intervention* and sex; *online child intervention* and parental support; *work continuance* and parental support; the presence of ID and age-group (preschooler or schooler). In order to take into consideration the time difference between T1 and T0 as a possible confounder or effect modifier variable, block regression analysis models were performed. The difference in ABAS_II scores between T1 and T0 was included as a dependent variable, while the presence of ID, sex, age, ADOS-CSS score, and the time difference between T1 and T0 were consecutively added as independent variables. For all multiple regression analyses, the dummy variable sex was coded as 0 = male and 1 = female and the dummy variable ID was coded as 0 = No ID and 1 = ID. An alpha level of 0.05 was used for all statistical analyses. Results are reported as means ± SDs if not otherwise specified. All statistical analyses were performed using SPSS v.23.0 (IBM Corp., Armonk, NY, USA).

## 4. Results

A total of 85 participants (age range 2–18 years old; mean age 7 years old; 68 (80.0%) males; 17 (20.0%) females) were included in the study (Figure 1). According to age, we divided the sample into two groups—“preschooler” (*n* = 33; age range 2–5 years old) and “schooler” (*n* = 52; age range 6–18 years old). 

The T1 evaluation (after the compulsory lockdown, within a period range of May–July 2020) was performed at a mean age difference (T0–T1) of 8.3 months for the preschoolers and of 10.3 months for schooler participants. The median age differences were 8.0 and 9.0 months, respectively (Table 1).

### 4.1. Clinical Summary

#### 4.1.1. Preschooler Group

At baseline (in 2019, before COVID-19), the preschooler group was characterized by a mean age of 4 years old; 25 males, 8 females; 24 No ID and 4 ID (five participants did not complete the cognitive evaluation); a median ADOS-CSS of 6.5 (indicating a moderate level of autistic symptoms severity) (Table 1). During the lockdown, among the preschoolers, 14 underwent an online intervention; as for their parents, 10 received online support and 15 preserved their job (either remotely or in-person) (Table 1). Mean ± SD for all outcome measures (ABAS-II, RBS, and CBCL) at T0 and T1 are reported in the Supplementary Materials (Table S3).

#### 4.1.2. Schooler Group

The schooler group presented a mean age of 9 years old; 43 males, 9 females; 29 No ID, 22 ID (one participant did not complete the cognitive evaluation), a median ADOS-CSS of 7 (indicating a moderate level of autistic symptoms severity). During the lockdown, within the schooler group, 24 ASD participants underwent an online intervention; 19 parents were supported online, and *work continuance* was reported by 30 parents (Table 1). Mean ± SD for all outcome measures (ABAS-II, RBS, and CBCL) at T0 and T1 are reported in the Supplementary Materials (Table S3).

### 4.2. T0–T1: Relation between Time Distance and Adaptive Functioning Results

The primary aim of this study was to investigate any change in adaptive functioning and in repetitive and behavioral (internalizing and externalizing) problems, appearing after the compulsory home confinement related to COVID-19. However, as described in the Methods section, in this observational study the time-interval between T0 and T1 varied between individuals. Therefore, before analyzing the observed paired differences, we had to investigate whether the distance between T0 (before COVID-19) and T1 (after lockdown) might have played a role either as a confounder or as an effect modifier on the adaptive skills findings (ABAS-II). The inclusion of T1–T0 time difference as a predictor in any linear regression model, where the difference in ABAS_II scores between T1 and T0 was considered as dependent variable and presence of ID, sex, age, and ADOS-CSS score were consecutively added as independent variables, did not reach statistical significance (Beta = −0.119; *p* = 0.805) and did not modify the beta coefficients of the other independent variables. Furthermore, after performing a Spearman correlation between “participants age difference T0–T1” and the “ABAS-II differences,” no significant results emerged (GAC: *p* = 0.962, PAD: *p* = 0.883, SAD: *p* = 0.637, CAD: *p* = 0.872). Moreover, we did not find statistically significant results when comparing “mean age differences T0–T1 of preschoolers” with “mean age differences T0–T1 of schoolers” (*t* = 1.951; *p* = 0.054), meaning that the two groups did not significantly differ in terms of time distance T0–T1. Finally, no significant difference emerged in terms of T0–T1 distance between participants with ID and without ID in both schoolers (*t* = 1.087; *p* =.282) and preschoolers (*t* = 0.155; *p* = 0.878).

### 4.3. Paired Differences between T0 (before COVID-19) and T1 (after the End of Lockdown): Adaptive Functioning

Within the preschooler group, after the lockdown, a significant improvement emerged in almost all the ABAS-II domains (Mean differences between T1 and T0: GAC = 11.07 ± 21.78, *t* =2.64, *p* = 0.014; CAD= 9.07 ± 20.68, *t* = 2.27, *p* = 0.031; PAD = 9.29 ± 23.20, *t* = 2.08, *p* = 0.047), except for the SAD (5.92 ± 18.90, *t* =1.62, *p* = 0.115), where no significant results were found (Table 2). In contrast, in the schooler group, no significant result was found between baseline and T1 in all the investigated adaptive domains (GAC = 0.78 ± 9.02, *t =* 0.58, *p* = 0.559; CAD = 1.22 ± 8.54, *t* = 0.95, *p* = 0.343; PAD = 0.37 ± 13.47, *t* = 0.18, *p* = 0.853; SAD = 0.522 ± 8.46, *t* = 0.41, *p* = 0.678) (Table 2).

### 4.4. Adaptive Skills: Relation to Child and Parental Variables within the Preschooler Group

Given the finding of a significant improvement in adaptive skills among the preschooler participants, we evaluated if child variables (presence or not of *intellectual disability; online intervention* during COVID-19) and parental variables (*work continuance* and *online parental support* during COVID-19) were related to the improvement observed at T1 (Table 3). 

#### 4.4.1. Child Variables

The improvement in the GAC domain that emerged within the preschooler group was not related to sex. It was instead significantly related to the absence of ID (*M =* 16.7 ± 23.5; *t* = 2.4; *p* = 0.023) (Table 3). Individuals with ASD without ID presented a GAC score improvement of 16.74 in comparison to their peers with ID who reported a score increase of 3 points (*M =* 3.0 ± 3.0). Moreover, the presence of an adequate intellectual quotient was also significantly related to the improvement in the social and practical adaptive domains of the investigated adaptive skills (Mean GAC *=* 11.0 ± 20.0; *t* = 1.1; *p* = 0.039; PAD = 15.7 ± 24.0; *t* = 2.3; *p* = 0.037) (Table 3).

In regards to the *online child intervention* during the lockdown, we found a significant improvement in the mean GAC (14.72 ± 21.21; *p* = 0.044) within individuals with ASD who received an online intervention (Table 3). Among the ASD participants not undergoing treatment during the lockdown, no significant improvement emerged in adaptive functioning (GAC = 8.56 ± 22.50; *p* = 0.149).

#### 4.4.2. Parental Variables

We found that participants whose parents underwent *online parental support* during the lockdown, had a significant improvement in the mean practical adaptive domain (PAD = 5.37 ± 5.44; *p* = 0.027) (Table 3), as opposed to the individuals with ASD whose parents did not receive such support (PAD = 11 ± 26; *p* = 1.1). Considering the variable *parental work continuance* during the lockdown, a significant improvement in the GAC (15.00 ± 25.77; *p* = 0.034), in the CAD (13.62 ± 25.24; *p* = 0.047) emerged amongst the ASD individuals with parents not continuing their usual job (Table 3). In contrast, the social domain (SAD) did not report any significant results (7.75 ± 22.78; *p* = 0.194) (Table 3). Alternatively, parents who continued working (either remotely or in-person) did not report a significant improvement in their child’s functioning (GAC = −5.36 ± 13.29; *p* = 0.211).

### 4.5. Adaptive Skills: Relation to Child and Parental Variables within the Schooler Group

Even if no significant difference in the adaptive skills emerged within the schooler sample after the lockdown, we investigated also in this group if child variables (presence or not of *Intellectual Disability; online intervention* during COVID-19) and parental variables (*work continuance* and *online parental support* during COVID-19) were related to these findings (Table 3).

#### 4.5.1. Child Variables

No significant relation emerged, within schoolers, between ABAS-II differences (before COVID-19 and after lockdown) and *online child intervention* (Table 3). In particular, undergoing online treatment during lockdown (ABAS_GAC: *t* = 0.609; *p* = 0.946) or not undergoing online treatment (ABAS_GAC: *t* = 1.7; *p* = 0.11) was not significantly related to adaptive functioning. Moreover, an IQ ≤ 70 (ID) or an IQ > 70 (No ID) was not significantly related to the differences in adaptive skills (ID: ABAS_GAC: *t* = 0.060, *p* = 0.953; No ID: ABAS_GAC *t* = 0.734, *p* = 0.47) (Table 3).

#### 4.5.2. Parental Variables

No significant relation emerged, within schoolers, between ABAS-II differences and both parental variables, *work continuance* and *online parental support* during COVID-19 (Table 3). Therefore, the fact of receiving an online support (ABAS_GAC: *t* = 1.4; *p* = 0.18) or not (ABAS_GAC: *t* =2; *p* = 0.056) and whether parents continued their usual job (ABAS_GAC: *t* = 1.2; *p* = 0.24) or not (ABAS_GAC: *t* = 0.24; *p* = 0.81) was not significantly related to the ABAS-II differences after lockdown due to COVID-19.

### 4.6. Paired Differences between T0 (before COVID-19) and T1 (after the End of Lockdown): Repetitive and Problematic Behavior

With regards to repetitive and problematic behaviors measured by RBS-R and CBCL, no significant results emerged between baseline and post-lockdown in both preschooler (RBS-R_Tot: *t* =1.3; *p* = 0.182; CBCL Tot: *t* = 0.83; *p* = 0.412) and schooler participants (RBS-R_Tot: *t* = 0.18; *p* = 0.853; CBCL Tot: *t* = 0.92; *p* = 0.363) (Table 2). Therefore, parents reported no improvement or worsening in these behavioral domains.

### 4.7. Interaction Analyses

A further aim of the study was to evaluate if the possible paired differences were associated with child and parental variables such as subsistence of child therapeutic intervention in remote modality, *online parental support*, and *parental work continuance* during home confinement. In this context, we had to consider also the possibility of heterogeneity of the effects of these interventions, i.e., intervention effects varying in relation to T0 characteristics. Therefore, in order to evaluate possible interactions between explanatory variables with regards to the adaptive skills improvement (ABAS_GAC difference T1–T0), we performed two-way ANOVA. When considering *cognitive* and *sex* as independent variables, no significant interaction was found (F(1.64) = 0.609; *p* = 0.44; Partial Eta Squared = 0.009). No significant results emerged when investigating the interaction between sex and *children online intervention* (F(1.66) = 2.966; *p* = 0.09; Partial Eta Squared = 0.043). Moreover, *children online intervention* and *online parental support* did not show a significant interaction too (F(1.66) = 0.038; *p* = 0.85; Partial Eta Squared = 0.001). Finally, no significant findings emerged in the interaction between *online parental support* and *work continuance* (F(1.66) = 1.775; *p* = 0.19; Partial Eta Squared = 0.026).

## 5. Discussion

In this study, we investigated the possible impact of the COVID-19 lockdown on the adaptive functioning, and the problematic and repetitive behaviors of a sample of ASD Italian preschoolers and schoolers. In particular, we evaluated whether after the compulsory home confinement, and in comparison to a baseline evaluation performed during the months preceding the pandemic, (in the context of regular clinical follow-up) any worsening or improvement was reported by parents of ASD individuals.

Interestingly, following the lockdown, in the re-opening phase, we did not find any worsening in the areas explored within preschooler and schooler participants. A significant improvement emerged in reference to adaptive functioning only within preschoolers, whereas substantial clinical stability in behavioral aspects (repetitive and problematic) was reported by parents belonging to both age groups.

### 5.1. Impact on Adaptive Functioning

After the end of home confinement, all the adaptive domains were reported as enhanced by parents of preschooler group children, except for the social domain, which did not improve. Given that during the lockdown as conducted in Italy, home exiting was forbidden and social relationships precluded, this result is not surprising. On the contrary, for the schooler group, clinical stability referring to adaptive skills was reported by the parents. The lack of significant improvement in adaptive functioning among the schoolers could derive from the fact that there was a major representation of individuals with ID as opposed to the preschooler sample. As a matter of fact, when investigating the possible variables related to a better outcome after the lockdown in the ASD preschooler group, we found a greater improvement in ASD participants without ID compared to their autistic peers with ID. This is consistent with the literature reporting a worse outcome in individuals with ASD and affected with cognitive impairment, although not under the conditions of home confinement [17,18].

Furthermore, we investigated the role of the behavioral intervention among the variables that may have influenced the positive impact that emerged on the adaptive functioning of ASD preschoolers. In fact, even if the in-person delivered intervention was interrupted during the lockdown, some individuals with autism received a temporary replacement by an online intervention. In particular, it is worth mentioning that, in ASD preschoolers, children who continued the behavioral intervention during lockdown with a frequency of at least once a week, reported a significant amelioration in the GAC (a comprehensive domain of the adaptive skills), which was a trend in all other adaptive domains (except for the social domain) and opposed to the ASD preschoolers who did not receive the web-delivered intervention.

Moreover, given the essential role of parental care in the interventions addressed to persons with autism [19,20], we explored the possible effects that parental variables occurring during lockdown (*online parental intervention*; *work continuance*) may have had on the adaptive functioning improvement that emerged. Specifically, we found that, in preschoolers, ASD children whose parents received online support during the lockdown, showed a significant improvement in the practical adaptive domain (PAD), which evaluates self-care, safety, home life, and care of the environment. This result may be explained by the possible role of online training in implementing parental strategies to improve practical skills at home during this period of lockdown.

Recent studies demonstrated that web-delivered psychoeducational programs addressed to both ASD youths and their parents have proven effectiveness in boosting skills during transition-age [21,22,23]. In fact, nowadays, telehealth interventions (teletherapy, telesupport) represent promising models for individuals with ASD, providing easy access to services otherwise not available in extreme circumstances, such as the lockdown has been [24,25].

Of special interest, our study highlights the positive and beneficial effects of the parental presence at home and in particular, of the time spent with children. In fact, parents who continued working during lockdown (either remotely or in-person) did not report any improvement in their sons’ and daughters’ functioning. In contrast, the group of autistic children whose parents did not continue their usual job (no *work continuance*), registered a significant amelioration in the adaptive skills (GAC, CAD, and a trend in the PAD). We presume that the parents who did not persist working during the lockdown, being constrained to a homestay by State’s regulation, spent more time with their children in comparison to the months preceding COVID-19, with a subsequent positive impact on the children’s functioning. Instead, we presume that the parents who maintained their usual job (either remotely or in-person) did not significantly implement the time spent with their children, thus resulting in a lack of positive effect on the children’s skills.

These findings suggest and underline the importance of parental care in ASD treatment, pertaining to involvement in the intervention and time spent at home with the children [19]. Moreover, the results of this study yield the recommendations of supporting parents through those specific services that may turn out helpful in improving skills learned within a therapeutical context. National health systems should therefore provide education to all families of individuals with ASD in emergency circumstances and in everyday life.

### 5.2. Impact on Behavioral Problems and Repetitive Behaviors

Lastly, we found substantial clinical stability in the level of repetitive and problematic behaviors in both groups. Our results concerning clinical stability for these behavioral features are not concordant with the behavioral problem increase reported during COVID-19 by Colizzi et al. [6] and Mutluer et al. [7].

In particular, Colizzi et al. conducted a parent survey on an Italian ASD pediatric sample. However, the measurement employed by the authors in order to measure these behavioral features did not rely on valid standardized tools similar to the ones we employed, but on a generic survey. Above all and in addition to this fact, no comparison with an equal evaluation performed before the COVID-19 pandemic was made. Therefore, in the study by Colizzi et al., the behavioral worsening reported by parents does not constitute a reliable measurement of the post-COVID-19 outcome but a general parental estimate of the present clinical picture. Moreover, demographic characteristics of the samples (mean age, region) could explain the non-concordant results between our (mean age 7 years old; central-south Italy) and the study by Colizzi et al. (mean age 13 years old; north of Italy) [6].

On a Turkish sample of 87 ASD individuals (3–29 years old) [7], the authors found an increase in behavioral problems measured by the parental questionnaire aberrant behavior checklist (ABC). Although employing a standardized measure, authors asked parents during the pandemic to answer in reference to both before and after the COVID-19 measures.

Furthermore, the feature that emerged in our study regarding the lack of a worsening in repetitive and problematic behavior could be explained by the fact that the sample population was originating from the central-south of Italy—one of the least COVID-19 affected regions of the Country, with restriction rules applied in a second step if compared to the north of Italy. Therefore, we hypothesize that for the individuals with autism taken into consideration in our study, the compulsory lockdown may have represented a less challenging period (shorter duration, lesser restriction, less stressed family environment) with a contextual non-significant enhancement of these dysfunctional behaviors.

On the other hand, the lack of improvement emerging from our study in reference to repetitive and problematic behaviors could be explained by the fact that such behaviors necessitate intensive intervention due to their pervasiveness and persistence [26,27,28]. Intervention that was not possible to undertake during the lockdown.

## 6. Strengths and Limits of the Study

One of the main strengths of our study is represented by the T1 and T0 evaluation of ASD participants with standardized tools, which allows us to measure the possible impact of the lockdown.

Noteworthy is the finding that, even if in our study the time–distance between T0 (before COVID-19) and T1 (after lockdown) varied between participants (see Methods section), we did not find any significant influence of this interval on the adaptive skills findings.

However, our research is characterized by several limitations, including the reduced size of the sample, in particular of the preschooler group manifesting a scarce representation of ASD individuals with ID; the employment of parental report measures, which do not offer an objective evaluation; studying a convenience sample (ASD children clinically followed by our unit were included in the study) in which no sample size calculation was performed in advance; and, being an observational study in which no homogeneity exists in the modality (duration, frequency) and contents of the web-delivered behavioral intervention and *online parental support*. Finally, the limited sample size and, therefore, the limited power of the study has possibly reduced our ability to detect heterogeneity in the intervention effects; although we did not find significant interactions, our results can neither confirm the absence of interactions nor that the observed outcomes necessarily apply to all subjects. Yet, we reported the performed subgroup even if it was not the primary aim of this study. Nevertheless, due to these limitations, the results of the paired analysis should be considered with caution.

## 7. Conclusions

Our research leaves open questions. In fact, we investigated the short-term impact of lockdown on behavior and adaptive functioning but we could not look into the long-term effects. However, having used standardized instruments allows us to replicate our findings even at a greater distance. Future studies on the topic are necessary in order to better understand and delineate the possible impact that the COVID-19 pandemic may have on the functioning of individuals with ASD and their families.

## Figures and Tables

**Table 1:** Main characteristics of the age groups.

	Age at Baseline (M ± SD)	Age Difference T0–T1 (M ± SD)	No ID-ID	ADOS 2-CSS (Median)	*Online* *Child Intervention*	Parental Support	*Work Continuance*
**PRESCHOOLER** **(*n* = 33;** **25 males; 8 females)**	52.3 ± 11.5 months	8.3 ± 4 months	24 (NoID)- 4 (ID) *	6.5	14	10	15
**SCHOOLER** **(*n* = 52;** **43 males; 9 females)**	110.1 ± 42.2 months	10.3 ± 4.8 months	29 (NoID)- 22 (ID) *	7	24	19	30

Shown in the table are the main clinical characteristics (age, cognitive ability, and autism severity); child and parental variables of the two age groups: preschooler and schooler. ADOS 2-CSS = Autism Diagnostic Observation Schedule-(Second Edition) calibrated severity score. Legend: ID = intellectual disability. No ID = no intellectual disability. * five children did not complete the IQ evaluation at baseline in the preschooler group; one child in the schooler group.

**Table 2:** Paired differences between T1 (after the end of lockdown; re-opening phase) and T0 (before COVID-19).

	Mean Difference T1–T0 (M ± SD)	*t*	*p* Value
PRESCHOOLER			
ABAS-II_GAC T1–T0	11.07 ± 21.78	2.64	0.014 *
ABAS-II_CAD T1–T0	9.07 ± 20.68	2.27	0.031 *
ABAS-II_SAD T1–T0	5.92 ± 18.90	1.62	0.115
ABAS-II_PAD T1–T0	9.29 ± 23.20	2.08	0.047 *
CBCL_INT T1–T0	−2.67 ± 6.97	1.87	0.074
CBCL_EXT T1–T0	−0.625 ± 7.15	0.43	0.673
CBCL_TOT T1–T0	−1.21 ± 7.08	0.83	0.412
RBS_TOT T1–T0	3.12 ± 11.35	11.37	0.182
SCHOOLER			
ABAS-II_GAC T1–T0	0.78 ± 9.02	0.58	0.559
ABAS-II_CAD T1–T0	1.22 ± 8.54	0.95	0.343
ABAS-II_SAD T1–T0	0.522 ± 8.46	0.41	0.678
ABAS-II_PAD T1–T0	0.37 ± 13.47	0.18	0.853
CBCL_INT T1–T0	−1.06 ± 7.54	0.82	0.419
CBCL_EXT T1–T0	0.00 ± 6.91	0.00	1.00
CBCL_TOT T1–T0	1.00 ± 6.32	0.92	0.363
RBS-R_TOT T1–T0	0.37 ± 12.71	0.18	0.853

ABAS-II = Adaptive Behavior Assessment System (Second Edition); GAC = general adaptive composite score; CAD = conceptual adaptive domain; SAD = social adaptive domain; PAD = practical adaptive domain; CBCL = child behavior checklist; CBCL_INT = CBCL_ internalizing symptoms; CBCL_EXT= CBCL_ externalizing symptoms; CBCL_TOT= CBCL total score; RBS-R_TOT= Repetitive Behavior Scale-Revised total score; * = significant value.

**Table 3:** Improvement in adaptive skills: relation to parental and child variables within the preschooler and schooler group.

	Mean Difference T1–T0 (M ± SD)	*t*	*p* Value
CHILD VARIABLES: *Children Undergoing Online* *Intervention during COVID-19 lockdown*			
ABAS-II_GAC T1–T0			
*Preschooler*	14.72 ± 21.21	2.3	0.044 *
*Schooler*	−0.13 ± 9.05	0.07	0.946
ABAS-II_CAD T1–T0			
*Preschooler*	13.18 ± 22.12	1.9	0.07
*Schooler*	−0.2 ± 9.1	1.1	2.99
ABAS-II_SAD T1–T0			
*Preschooler*	8.00 ± 19.63	1.3	0.20
*Schooler*	1.1 ± 7.6	0.71	0.485
ABAS-II_PAD T1–T0			
*Preschooler*	12.27 ± 22.00	1.8	0.09
*Schooler*	−0.35 ± 11.2	0.15	0.884
*Participants with IQ > 70 (No ID)*			
ABAS-II_GAC T1–T0			
*Preschooler*	16.7 ± 23.5	2.4	0.023 *
*Schooler*	1.5 ± 10.4	0.734	0.47
ABAS-II_CAD T1–T0			
*Preschooler*	13.9 ± 22.5	1.8	0.089
*Schooler*	3.1 ± 10.25	1.5	0.15
ABAS-II_SAD T1–T0			
*Preschooler*	11 ± 20	1.1	0.039 *
*Schooler*	1.0 ± 7.8	0.5	0.96
ABAS-II_PAD T1–T0			
*Preschooler*	15.7 ± 24	2.3	0.037 *
*Schooler*	1.0 ± 16.23	0.31	0.761
PARENTAL VARIABLES: *Parents Receiving Online* *Support during COVID-19 lockdown*			
ABAS-II_GAC T1–T0			
*Preschooler*	7.2 ± 10.23	2.00	0.085
*Schooler*	1.8 ± 5.6	1.40	0.180
ABAS-II_CAD T1–T0			
*Preschooler*	5.1 ± 9.92	1.46	0.188
*Schooler*	6.1 ± 5.41	0.479	0.638
ABAS-II_SAD T1–T0			
*Preschooler*	3.50 ± 12.41	0.79	0.457
*Schooler*	0.00 ± 7.56	0.000	1.00
ABAS-II_PAD T1–T0			
*Preschooler*	5.3 ± 5.44	2.79	0.027 *
*Schooler*	1.94 ± 9.3	0.887	0.387
*Parents not continuing their usual job* *during COVID-19 lockdown*			
ABAS-II_GAC T1–T0			
*Preschooler*	15.00 ± 25.77	2.3	0.034 *
*Schooler*	−0.500 ± 7.27	0.24	0.812
ABAS-II_CAD T1–T0			
*Preschooler*	13.62 ± 25.24	2.1	0.047 *
*Schooler*	−0.78 ± 7.3	0.40	0.693
ABAS-II_SAD T1–T0			
*Preschooler*	7.75 ± 22.78	1.3	0.194
*Schooler*	9.3 ± 9.43	0.36	0.719
ABAS-II_PAD T1–T0			
*Preschooler*	12.43 ± 27.27	1.8	0.088
*Schooler*	−0.57 ± 5.5	0.38	0.704

Shown in the Table are the impact of the child (children receiving online intervention during the lockdown, children without intellectual disability) and parental variables (parents receiving *online parental support*; parents not continuing their usual job during the lockdown either in person or remotely) on adaptive skills the difference T1–T0. Legend: ABAS-II = Adaptive Behavior Assessment System (Second Edition); GAC = general adaptive composite score; CAD = conceptual adaptive domain; SAD = social adaptive domain; PAD = practical adaptive domain; * = significant value.

## Data Availability

The data presented in this study are contained within the article and supplementary material.

## Supplementary Materials

The following are available online at https://www.mdpi.com/2227-9067/8/2/96/s1, Table S1: Study Protocol, Table S2. Strobe Checklist, Table S3: ABAS-II, CBCL and RBS subscales scores at T0 and T1 in preschooler and schooler group.
