# Supplementary material for: The Impact of COVID-19 on the Adaptive Functioning, Behavioral Problems, and Repetitive Behaviors of Italian Children with Autism Spectrum Disorder: An Observational Study
Source: Children (Basel). 2021 Feb 2;8(2):96. doi: 10.3390/children8020096 (PMC7913091; doi:10.3390/children8020096)
Supplement: Supplementary file 1 [file children-08-00096-s001.pdf]

Supplementary Material

Table S1. Study Protocol.

**Recruitment (March-April 2020)**
A multidisciplinary team of psychiatrists and psychologists of the *Child Psychiatry Unit of the Tor Vergata University Hospital of Rome,* examined the clinical database of the Unit and considered all the included patients for eligibility. 335 individuals with Autism Spectrum Disorder were detected from the database; 208 did not meet inclusion criteria. The team contacted the families by phone, described the study and invited them to participate planning a subsequent telehealth appointment, 8 declined to participate. 119 were included in the study. Parents of all participants provided informed consent.<br>34 skipped the telehealth appointment and dropped out the study. Therefore the final sample consisted of 85 participants (80% males; 20% females; age range 2-18 yrs; 33 preschoolers, 52 schoolers)
**Tele-Health appointment (May -July 2020)**
After the compulsory home-confinement, from May to July 2020, in the re-opening phase (T1), ASD children included in the study and their parents underwent a planned tele-health appointment with a child psychiatrist of the Rome Tor Vergata University Hospital.<br>During the tele-health appointment, parents of ASD participants were clinically interviewed in order to evaluate the main routine disruption and the environmental changes occurred during the pandemic. Specifically, the following variables were investigated: whether during lockdown ASD children continued their usual behavioral intervention in remote modality and with a frequency of at least once a week (variable named *online child intervention)*; whether parents received at least a weekly online psychoeducational support in order to be helped face their children's main and overall difficulties due to the emergency situation (variable named *online parental support*); if the parent with a stable job, continued to be employed during the lockdown, either remotely or in-person (variable named *work continuance)*.<br>Moreover, the questionnaires ABAS-II, RBS-R, CBCL were administered to the parents.
**Comparison between clinical evaluation performed in 2019 –before COVID 19 outbreak - (T0) and after lockdown (T1)**
Results of all the ABAS-II, RBS-R, CBCL questionnaires administered to parents of ASD participants during the tele-health appointment (T1) were compared with the respective questionnaires performed before the COVID-19 outbreak in the context of regular clinical follow-up (T0).

Table S2. Strobe Checklist.

	**Item No**	**Recommendation**	**Endorsed**	**Page**
**Title and abstract**	1	(*a*) Indicate the study's design with a commonly used term in the title or the abstract	Y	1
		(*b*) Provide in the abstract an informative and balanced summary of what was done and what was found	Y	1
**Introduction**				
Background/rationale	2	Explain the scientific background and rationale for the investigation being reported	Y	2
Objectives	3	State specific objectives, including any prespecified hypotheses	Y	2
**Methods**				
Study design	4	Present key elements of study design early in the paper	Y	3
Setting	5	Describe the setting, locations, and relevant dates, including periods of recruitment, exposure, follow-up, and data collection	Y	3, "*Procedure*" section
Participants	6	(*a*) Give the eligibility criteria, and the sources and methods of selection of participants. Describe methods of follow-up	Y	3, "*Participants"* section
		(*b*) For matched studies, give matching criteria and number of exposed and unexposed	NA	
Variables	7	Clearly define all **outcomes**, exposures, predictors, **potential confounders**, and effect modifiers. Give **diagnostic criteria**, if applicable	Y	3-4 "*Participants*" and "*Materials*" section
Data sources/ measurement	8	For each variable of interest, give sources of data and details of methods of assessment (measurement). Describe comparability of assessment meth-	Y	4-5 *"Materials"* section

		ods if there is more than one group		
Bias	9	Describe any efforts to address potential sources of bias	N	
Study size	10	Explain how the study size was arrived at	NA	
Quantitative variables	11	Explain how quantitative variables were handled in the analyses. If applicable, describe which groupings were chosen and why	Y	3, "*Procedure*" section
Statistical methods	12	(*a*) Describe all statistical methods, including those used to control for confounding	Y	5-6, "*Statistical Analyses"* section
		(*b*) Describe any methods used to examine subgroups and interactions	Y	5-6, "*Statistical Analyses"* section
		(*c*) Explain how missing data were addressed	N	
		(*d*) If applicable, explain how loss to follow-up was addressed	NA	
		(*e*) Describe any sensitivity analyses	NA	
**Results**				
Participants	13	(a) Report numbers of individuals at each stage of study—eg numbers potentially eligible, examined for eligibility, confirmed eligible, included in the study, completing follow-up, and analysed	Y	3, "*Participants*" section; Figure 1
		(b) Give reasons for non-participation at each stage	NA	
		(c) Consider use of a flow diagram	Y	Figure 1
Descriptive data	14	(a) Give characteristics of study participants (eg demographic, clinical, social) and information on exposures and potential confounders	Y	Table 1
		(b) Indicate number of participants with missing data for each variable of interest	N	
		(c) Summarise follow-up time (eg, average and total amount)	NA	
Outcome data	15	Report numbers of outcome events or summary measures over time	N	
Main results	16	(*a*) Give unadjusted estimates and, if applicable, confounder-adjusted estimates and their precision (eg, 95% confidence interval). Make clear which confounders were adjusted for and why they were included	Y	8-13, "*Results*" section<br>We performed a stepwise linear regression analisys with ABAS_GAC difference as dependent variable and several factors (sex, age, ADOS CSS and T0-T1 time distance) as stepwise entered independent variables; we observed that the regression coefficient of intellectual ability did not change.
		(*b*) Report category boundaries when continuous variables were categorized	NA	
		(*c*) If relevant, consider translating estimates of relative risk into absolute risk for a meaningful time period	NA	

Other analyses	17	Report other analyses done—eg analyses of subgroups and interactions, and sensitivity analyses	Y	8-13, "*Results*" section
**Discussion**				
Key results	18	Summarise key results with reference to study objectives	Y	13
Limitations	19	Discuss limitations of the study, taking into account sources of potential bias or imprecision. Discuss both direction and magnitude of any potential bias	Y	15
Interpretation	20	Give a cautious overall interpretation of results considering objectives, limitations, multiplicity of analyses, results from similar studies, and other relevant evidence	Y	13-15 "*Discussion*" and "*Strenghts and limitation*" sections
Generalisability	21	Discuss the generalisability (external validity) of the study results	N	
**Other information**				
Funding	22	Give the source of funding and the role of the funders for the present study and, if applicable, for the original study on which the present article is based	NA	

Legend: NA= Not applicable; Y=yes; N= no.

Table S3. ABAS-II, CBCL and RBS subscales scores at T0 and T1 in preschooler and schooler group.

	**T0 (M ± SD)**	**T1 (M ± SD)**
**PRESCHOOLER**		
**ABAS-II_GAC**	56.7±11	67.8±25.7
**ABAS-II_CAD**	62.9±12.9	72±23.9
**ABAS-II_SAD**	63.5±11.5	69.4±22.6
**ABAS-II_PAD**	59.5±13.1	68.8±26.6
**CBCL_INT**	61.7±10.5	59±12.9
**CBCL_EXT**	58.1±12.4	57.5±11.5
**CBCL_TOT**	60.7±13	59.5±12.8
**RBS-R_STB**	5.1±5	5.2±4.8
**RBS-R_SIB**	1.1±1.6	0.5±0.8
**RBS-R_CB**	1.9±3.3	2.5±3.1
**RBS-R_RB**	4.9±4.9	6.7±5.8
**RBS-R_RIB**	1.9±2.3	2.4±2.1
**RBS-R_TOT**	15.1±13.3	18.2±13.2
**SCHOOLER**		
**ABAS-II_GAC**	55.6±15.3	56.4±15
**ABAS-II_CAD**	62.1±14.7	63.3±15.2
**ABAS-II_SAD**	65.2±14.4	65.7±12.8
**ABAS-II_PAD**	55.3±16.6	55.7±16.7
**CBCL_INT**	61.2±8.5	60.1±8.9
**CBCL_EXT**	56.7±10.2	56.7±10.2
**CBCL_TOT**	63.2±8.7	63.2±9.5
**RBS-R _STB**	6.6±5.2	7±6.9
**RBS-R_SIB**	2.1±3.3	1.8±3.5
**RBS-R_CB**	3.3±3.3	2.3±2.8
**RBS-R_RB**	6.1±5.6	6.7±6.9
**RBS-R_RIB**	2.7±2.2	2.7±2.2
**RBS-R_TOT**	20.9±14.3	20.5.±18.1

ABAS-II = Adaptive Behavior Assessment System, Second Edition; GAC = General Adaptive Composite score; CAD = Conceptual Adaptive Domain; SAD = Social Adaptive Domain; PAD = Practical Adaptive Domain; CBCL = Child Behavior Checklist; INT = Internalizing Symptoms; EXT= Externalizing Symptoms; TOT= total score; RBS-R = Repetitive Behavior Scale Revised; STB = Stereotypic Behavior; SIB = Self-injurious Behavior; CB= Compulsive Behavior; RB = Ritualistic Behavior; RIB = Restricted Interests Behaviors; TOT = total score.
